# One‐Pot Construction of NHS‐Activated Magnetic Particles for Chemoselective Capture of Carboxyl Metabolites

**DOI:** 10.1002/advs.202413830

**Published:** 2025-02-11

**Authors:** Shuai Liu, Meng Yu, Xin‐Yao Luo, Jie Liu, Zhong‐Mei Zou

**Affiliations:** ^1^ State Key Laboratory of Bioactive Substance and Function of Natural Medicines Institute of Medicinal Plant Development Chinese Academy of Medical Sciences & Peking Union Medical College Beijing 100193 China; ^2^ Tianjin University of Traditional Chinese Medicine Tianjin 300193 China

**Keywords:** functional materials, polymerization, SCFAs, versatile probes

## Abstract

Chemoselective probes immobilize on magnetic materials show great promise in simplifying sample handling and enhancing detection sensitivity. However, their complicated preparation and associated expense limit broader application. In this study, novel magnetic particles with abundant N‐hydroxysuccinimide (NHS) esters on the surface are conveniently synthesized using a one‐pot method without carbodiimide activation carboxylate molecules. Subsequently, multifunctional probes are synthesized by immobilizing high‐density chemical probes on the surface of the magnetic materials through a postsynthetic modification strategy. This versatile probe facilitates the rapid capture of carboxylated compounds from complex matrices, with the labeled metabolites release from the magnetic materials subsequently analyzed using ultra‐high performance liquid chromatography‐mass spectrometry (UHPLC‐MS). The advantages of this innovative chemical biological tool include the simplicity and low cost of the synthesis, as well as the capability to analyze polar and volatile carboxylated metabolites via LC‐MS. This new strategy is successfully applied to analyze short‐chain fatty acids (SCFAs) in rat cecal contents, demonstrating the reliability of the analytical method. This study presents a cost‐effective and easy‐to‐implement approach for preparing NHS‐activated magnetic particles and offers a versatile probe with chemoselective extraction and labeling capabilities, providing a practical tool for analyzing SCFAs in the gut.

## Graphical abstract

A novel strategy for the one‐pot synthesis of NHS‐activated magnetic particles from isocyanates is proposed. These synthesized magnetic particles function as effective immobilization carriers for chemical probes. The probe conjugates demonstrate excellent separation in UPLC‐MS analyses, and the absence of matrix effects enable direct, high‐sensitivity analysis of short‐chain fatty acids (SCFAs) without further derivatization.

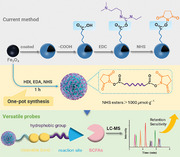

## 1 Introduction

Polymer materials are biocompatible, effective, and porous, and they can be constructed to possess specific functional properties. As a result, they have been extensively used for the separation and analysis of targeted molecules from mixed samples.^[^ ^1^ ^]^ The intrinsic magnetic properties of the magnetic nanoparticles (MNPs) facilitate convenient separation methods, such as magnetic decantation, eliminating the need for traditional centrifugation or flocculation typically required for the recovery of nonmagnetic materials.^[^ ^2^ ^]^ By introducing a shell of dendrimers or polymers to the surface of the MNPs, the diversity and loading of functional groups in magnetic polymer materials can be significantly enhanced.^[^ ^2^, ^3^, ^4^ ^]^ The employment of these functionalized magnetic polymer materials in biomedical research has achieved improved performance.^[^ ^5^ ^]^

Currently, it is straightforward and versatile to introduce functional groups such as ─OH, ─NH_2_ and ─COOH onto the surface of magnetic materials by coating MNPs with silicon dioxide (SiO_2_), polydopamine (PDA) and polyacrylic acid (PAA).^[^ ^6^ ^]^ However, it is important to note that controlling the spatial distribution of these functional groups can be challenging, and the reactivity of specific linkers, particularly hydroxyl groups, may be limited in some contexts.^[^ ^7^, ^8^, ^9^ ^]^ Moreover, it is hard to efficiently covalently conjugate biomolecules onto magnetic materials by postsynthetic modification strategy due to the steric hindrance. Chemoselective probe labeling strategies have greatly facilitated research in proteome^[^ ^10^, ^11^ ^]^ and genomics,^[^ ^12^, ^13^ ^]^ demonstrating tremendous potential for in‐depth description of metabolomics.^[^ ^14^, ^15^, ^16^ ^]^ Furthermore, chemical probes immobilized on magnetic materials provide a rapid and powerful analytical tool for recognizing metabolites in complex matrices.^[^ ^3^, ^17^, ^18^ ^]^ Therefore, preparing functionalized magnetic materials with abundant activated groups is essential for facilitating rapid postsynthetic modification and ensuring efficient extraction of target molecules.

N‐hydroxysuccinimide‐esters (NHS‐esters) are extensively used as functional groups in bioconjugation reactions, enabling the formation of amide bonds with amines.^[^ ^19^ ^]^ NHS‐esters exhibit relative stability and can be stored for months under dry conditions.^[^ ^20^ ^]^ NHS‐ester modifying reactions have broad availability and widespread utilization, such as protein labeling as well as surface activation of chromatographic supports, nanoparticles, and microarray slides.^[^ ^21^, ^22^ ^]^ NHS‐esters are reactive groups of carbodiimide activation carboxylate molecules, frequently in the presence of dicyclohexylcarbodiimide (DCC) and 1‐(3‐Dimethylaminopropyl)‐3‐ethylcarbodiimide (EDC).^[^ ^23^, ^24^ ^]^ Consequently, the availability of sufficient NHS‐esters on a solid material is significantly constrained by the density and spatial distribution of carboxyl groups. It is crucial to develop new strategies for the efficient preparation of these active esters that do not rely on standard coupling reactions involving carboxylic acids activated by carbodiimide reagents.

Our previous study reported the direct introduction of NHS‐esters on magnetic materials, but the reaction mechanism is not completely clear, and its low synthesis efficiency and uncontrollable synthesis process limit its expanded applications.^[^ ^4^ ^]^ In this paper, we present an original approach for facile and rapid synthesis of NHS‐activated polyurea‐coated magnetic microparticles (PMMPs) using a one‐pot method. Our study revealed that isocyanate can react with NHS in an addition reaction to generate NHS‐esters. To the best of our knowledge, this represents the first explicit report of a novel method for synthesizing NHS‐esters from isocyanate. In the synthesis of diamine‐diisocyanate polyurea, NHS, containing active N‐hydroxyl group, was added to enhance the control over the polymerization process and facilitate the direct synthesis of abundant NHS‐esters on the PMMPs. Additionally, multifunctional probes capable of chemoselectively capturing carboxylate metabolites were synthesized by systematically introducing functional groups onto the PMMPs through a post‐synthetic modification method. More importantly, these probes not only effectively capture carboxyl metabolites in complex matrix samples, but also yield SCFAs conjugated compounds with favorable hydrophobicity, allowing successful LC‐MS analysis and good chromatographic separation on a C18 column. The developed method was applied to analyze SCFAs in rat cecal contents, revealing significant differences in the levels of various SCFAs in fecal samples of control and model rats.

## 2 Results and Discussion

### 2.1 Preparation and Characterization of the Polyurea‐Coated Magnetic Microparticles (PMMPs)

**Scheme 1:**
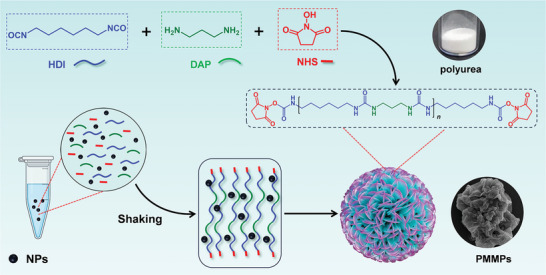
Schematic diagram of the preparation for NHS‐activated PMMPs.

The preparation procedure for PMMPs is schematically illustrated in **Scheme** **1**. Briefly, NHS, 1,3‐diaminopropane (DAP), and hexamethylene diisocyanate (HDI) were sequentially mixed in N, N‐dimethylformamide (DMF) solvent and stirred for 1 h at 25 °C. Then, the resulting mixture is subjected to centrifugation and freeze‐drying, yielding NHS‐activated polyurea in the form of a white powder. Additionally, NHS‐activated PMMPs were synthesized in a controlled manner by incorporating Fe_3_O_4_ nanoparticles (Fe_3_O_4_‐NPs) into the reaction mixture.

**Figure 1:**
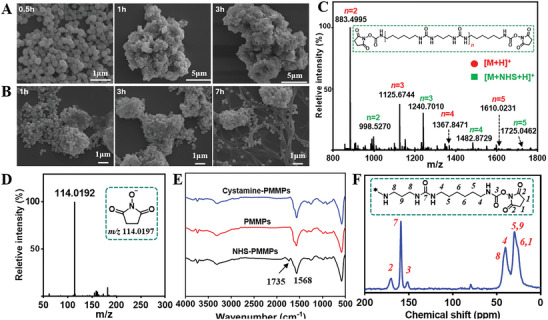
A) SEM images of the magnetic particles synthesized in DMF solvent at 0.5, 1, and 3 h. B) SEM images of magnetic particles synthesized in DMSO solvent at 1, 3, and 7 h. C) MALDI‐FTICR MS spectrum of polyurea particles. D) Mass spectra of NHS detected in the alkaline eluent of prepared polyurea particles or PMMPs. E) FTIR spectra of PMMPs with NHS‐activated moieties, PMMPs that released NHS in an alkaline solution, and cystamine‐modified PMMPs. F) Solid‐state ^13^C NMR spectrum of the polyurea particles.

To optimize the synthesis conditions of PMMPs, the reaction solvents acetonitrile (ACN), N,N‐dimethylformamide (DMF), and dimethyl sulfoxide (DMSO) were initially evaluated under synthesis conditions using a 1:1.5 molar ratio of DAP to HDI and a 0.1 m concentration of NHS. In ACN solution, the mixture of DAP, NHS, and HDI immediately produced white polymer precipitates, preventing the polymer from encapsulating Fe_3_O_4_‐NPs and forming a composite material. In contrast, the same mixture in a DMF solution yielded stable PMMPs with an approximate size of 10 µm and varying gaps within ≈1 h, and the polymer structure remained largely unchanged with extended reaction time (**Figure** **1**A). Whereas the Fe_3_O_4_‐NPs were only partially aggregated into clusters after 1 to 3 h of reaction in DMSO, polymer micelles were seen to be attached to the MNPs after 7 h (Figure 1B). The structures of polyurea were investigated using matrix‐assisted laser desorption‐fourier transform ion cyclotron resonance mass spectrometry (MALDI‐FTICR MS). As shown in Figure 1C, distinct peaks corresponding to polyurea with varying molecular weights were observed in positive ion mode. Additionally, peaks for [M+NHS+H]^+^ were detected concurrently, attributed to the release of unstable NHS‐esters from the polyurea due to freeze‐drying. The polyurea particles and PMMPs exhibited hydrolysis in aqueous or alkaline solvents, leading to the release of free NHS, but did not hydrolyze in organic solvents such as ACN or DMF, indicating that NHS moieties were covalently bonded to the polymers via hydrolyzable linkages (Figure 1D).

The FTIR spectra of PMMPs with NHS‐activated esters, PMMPs that released NHS in an alkaline solution, and cystamine‐modified PMMPs are shown in Figure 1E. The observed bands at 1735 and 1568 cm⁻¹ correspond to the C═O stretching vibration of NHS‐esters and the N─H bending vibration in polyurea, respectively. NHS‐esters on the polyurea were hydrolyzed and released in alkaline solvents, while the primary amine on cystamine rapidly replaced the NHS‐esters to form stabilized amide bonds, resulting in the disappearance of the 1735 cm⁻¹ peak in PMMPs with released NHS esters and cystamine‐modified PMMPs. Solid‐state ¹^3^C NMR spectroscopy was employed to further validate these findings. The spectra revealed peaks for the urea moieties in polyurea (151.46 ppm), the ester bond linking NHS to polyurea (158.98 ppm), and the carbonyl group of NHS moieties (170.24 ppm), with detailed peak assignments provided in Figure 1F.

### 2.2 Synthesis Design and Experimental Optimization

Due to the high nucleophilicity of primary amines, the reactivity of amines is much higher than that of water and primary alcohols in reaction toward isocyanate.^[^ ^25^, ^26^ ^]^ Consequently, the diamine‐diisocyanate polyurea synthesis readily occurs at room temperature and does not require the use of a catalyst.^[^ ^26^ ^]^ Under anhydrous conditions, the synthesis of diamine‐diisocyanate polyurea with excess diamine or diisocyanate is expected to yield magnetic polyurea composites with surfaces functionalized with amine or isocyanate groups. Attempts had been made to synthesize DAP‐HDI magnetic polyurea materials in DMF and DMSO solutions; however, the resulting products were difficult to clean and magnetically separate, hindering further investigation. Scanning electron microscope (SEM) images (Figure S1, Supporting Information) indicate that the polymer does not fully encapsulate the MNPs, with polymer micelles and PMMPs coexisting and adhering to one another.

**Figure 2:**
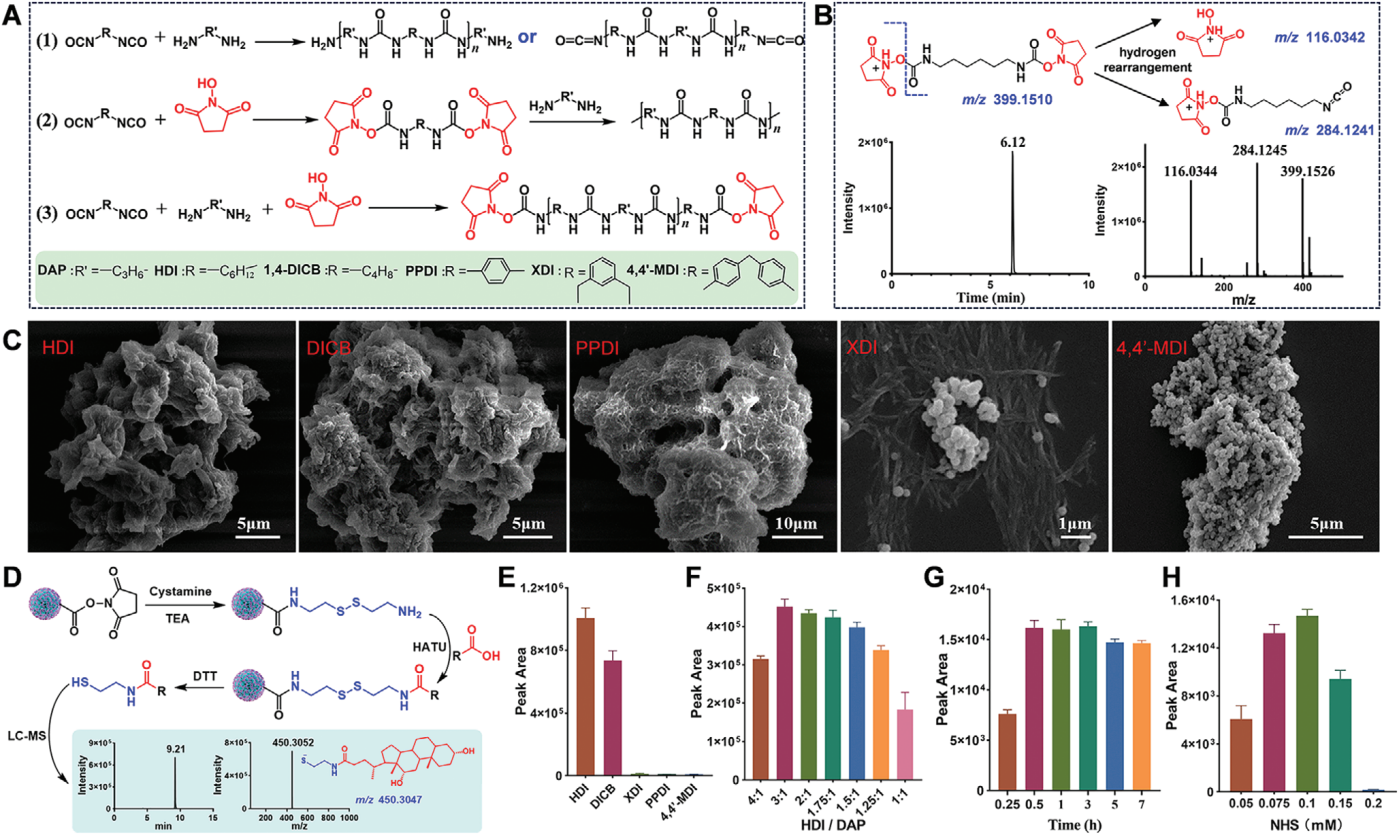
A) Chemical reactions involved in the polymerization of diamine and diisocyanate in a DMF solution containing NHS. B) LC‐MS spectra of the reaction products derived from NHS and HDI. C) SEM images of magnetic particles synthesized from five different diisocyanates in conjunction with DAP, NHS, and MNPs. D) Chemoselective extraction strategy employed for the analysis of carboxyl‐containing compounds using cystamine‐modified PMMPs. E) Peak areas of deoxycholic acid conjugates released from PMMPs prepared from five types of diisocyanates. Optimization of the DAP‐HDI polyurea synthesis, including the DAP/HDI ratio F), reaction time G), and NHS concentration H). Data are expressed as mean ± SD (*n* = 3).

NHS‐esters are typically synthesized by activating the carboxyl group using EDC/NHS in a two‐step reaction. The process involves: 1) the addition of the carboxylic acid to the carbodiimide group of the EDC molecule, resulting in the formation of an O‐acyl urea intermediate; and 2) the subsequent nucleophilic attack by NHS on the O‐acyl urea intermediate to form a relatively stable NHS ester (Figure S2, Supporting Information). In this study, LC‐MS analysis for the first time demonstrated that NHS reacts with HDI in DMF solution to produce NHS‐esters (**Figure** **2**A). Consequently, NHS plays a critical role in the synthesis of NHS‐activated PMMPs using DAP and HDI in this work. NHS competes with the diisocyanate‐diamine reaction by forming NHS‐esters with isocyanate groups, thereby decreasing the polymerization reaction rate and facilitating the controlled synthesis of PMMPs. Furthermore, as diamine is consumed, the excess NHS and diisocyanate ensure that a significant amount of NHS‐esters is attached to the surface of the synthesized PMMPs (Figure 2B). Five PMMPs were synthesized using three aliphatic diisocyanates and two aromatic diisocyanates to investigate their effects on the structure of the PMMPs and NHS esters density. As illustrated in Figure 2C, under the current reaction conditions, HDI, 1,4‐diisocyanatobutane (DICB) and 1,4‐phenylene diisocyanate (PPDI) produced PMMPs with a relatively sparse structure. Fibrous micelles synthesized by m‐xylylene diisocyanate (XDI) failed to encapsulate the MNPs to form PMMPs, while only adherent MNPs were observed in the products from 4,4′‐methylenediphenyl diisocyanate (MDI). The primary amine group of cystamine quickly reacts with the NHS‐esters on the PMMPs to form cystamine‐modified PMMPs, which can subsequently be further condensed with carboxylic compounds like deoxycholic acid using O‐(7‐azabenzotriazol‐1‐yl)‐N,N,N″,N″‐tetramethyluronium hexafluorophosphate (HATU). This process ultimately facilitates the reduction of disulfide bonds by dithiothreitol (DTT), leading to the rapid release of targeted analytes (Figure 2D). The deoxycholic acid conjugates released from the PMMPs were analyzed by LC‐MS. As shown in Figure 2E, the coupling modification efficiency of PMMPs synthesized with the aliphatic diisocyanates HDI and DICB was significantly higher than that of those synthesized with aromatic diisocyanates, with PMMPs synthesized from HDI exhibiting the best performance.

The study further explored the ratio of HDI to DAP, as well as the concentration of NHS and the reaction time. It was essential to investigate varying molar ratios of HDI to DAP due to HDI serves a dual role: it not only polymerizes with DAP but also reacts with NHS to form NHS‐esters. Consequently, molar ratios of HDI ranging from 1 to 4 times that of DAP were examined. As shown in Figure 2F, the peak areas of the targeted analytes increased as the molar ratio of HDI/DAP from 1 to 3, followed by a significant decrease at a molar ratio of 4. Therefore, a molar ratio of HDI/DAP of 3 was selected for the preparation of PMMPs. The duration of polymerization significantly influences both the morphology and capture efficiency of PMMPs. As shown in Figure 2G, PMMPs prepared for 0.5 h exhibited the highest capture efficiency, which remained stable up to 3 h before experiencing a slight decline with extended reaction time. Taking into account the robustness of the synthesis process and the reduced time requirements, a polymerization time of 1 h was determined to be optimal for the preparation of PMMPs. Additionally, the concentration of NHS plays a critical role in the synthesis of NHS‐activated PMMPs. Further investigation into NHS concentrations ranging from 0.05 to 0.2 m revealed that PMMPs synthesized with 0.1 m NHS demonstrated the highest capture efficiency (Figure 2H).

### 2.3 Characterization of PMMPs

**Figure 3:**
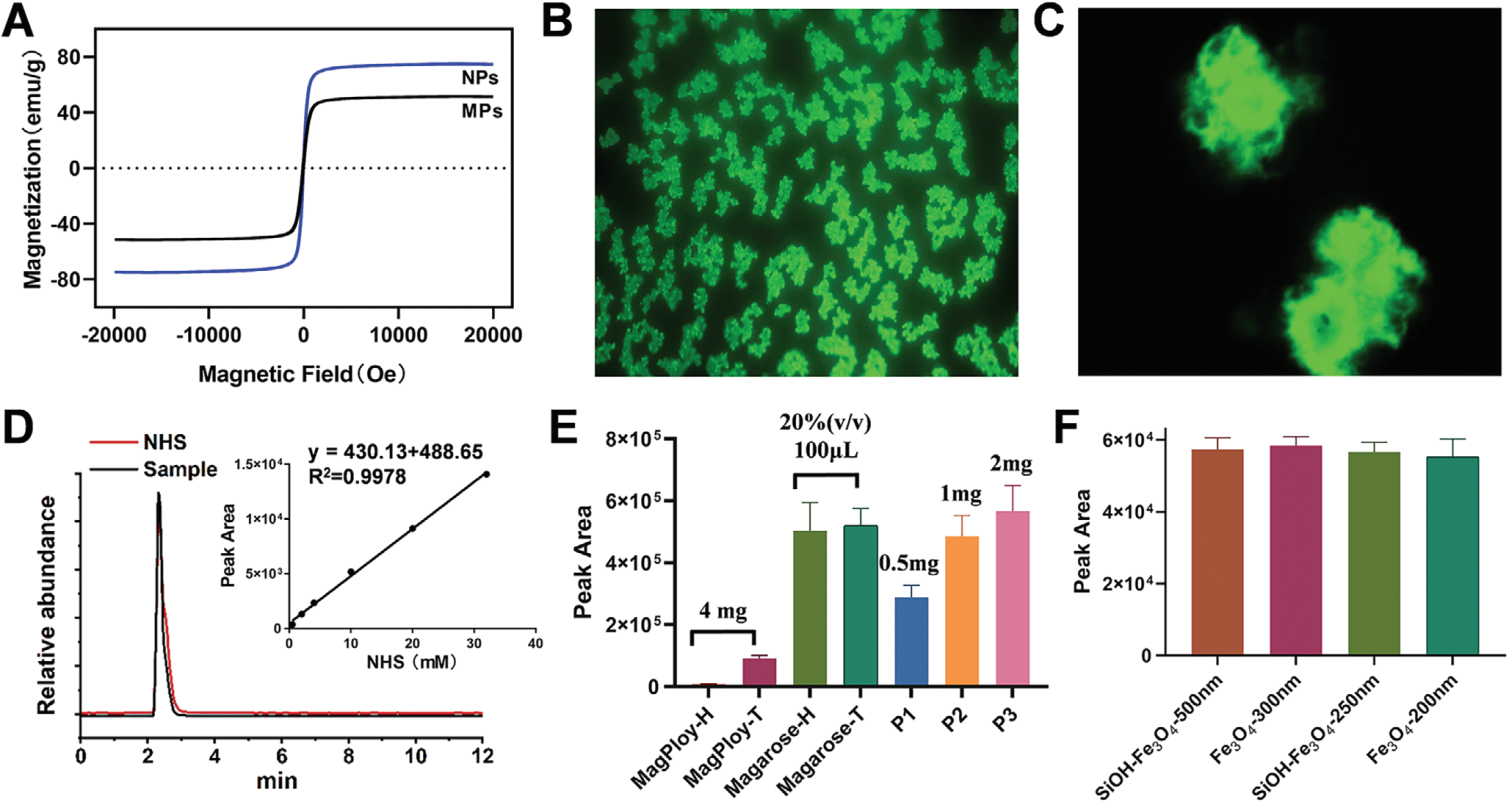
Characterization of PMMPs. A) Magnetic hysteresis curves for MNPs and PMMPs. B) Fluorescence microscopy of FITC‐labeled cystamine‐modified PMMPs. C) Laser confocal micrograph of FITC‐labeled cystamine‐modified PMMPs. D) Quantification of NHS‐esters on the PMMPs via LC‐MS analysis using a HILIC column. E) Comparative performance of PMMPs, NHS‐activated magnetic polymer microspheres (Magploy), and NHS‐activated magnetic agarose microspheres (Magarose) produced by H and T Corporation in the coupling of deoxycholic acid following cystamine modification. F) Performance evaluation of PMMPs derived from four types of MNPs in coupling deoxycholic acid after cystamine‐modified. Data are expressed as mean ± SD (*n* = 3).

The magnetic properties of the synthesized magnetic particles were assessed using a vibrating sample magnetometer (VSM). As illustrated in **Figure** **3**A, the saturation magnetization value of the Fe_3_O_4_‐MNPs produced via the hydrothermal method was measured at 75.0 emu g^−1^. After encapsulation with a polyurea layer, the saturation magnetization of the resulting PMMPs was reduced to 51.6 emu g^−1^. NHS‐esters are widely recognized as effective amine‐specific functional groups, frequently employed for cross‐linking and labeling primary amine ligands. The cross‐linking efficiency of NHS‐esters on solid substrates is typically influenced by factors such as NHS ester density, buffer pH, and steric hindrances related to surface interactions.^[^ ^27^ ^]^ NHS‐activated PMMPs can be efficiently coupled with cystamine dihydrochloride in 2‐morpholinoethanesulfonic acid (MES) buffer,^[^ ^28^ ^]^ where the primary amine group on the cystamine molecule reacts with the isothiocyanate group of fluorescein isothiocyanate (FITC) to conjugate the fluorescent marker to the PMMPs. Figure 3B shows a relatively uniform yellow‐green fluorescence at 520 nm from the FITC‐labeled PMMPs observed under the fluorescence microscope, indicating the good homogeneity of the synthesized PMMPs. Furthermore, laser confocal microscopy (Figure 3C) reveals a uniform distribution of fluorescence on the PMMP surface, suggesting that the spatial arrangement of NHS‐esters is homogeneous and capable of efficiently covalently binding target ligands.

The determination of free NHS represents a robust and versatile method for monitoring reactions involving NHS‐esters.^[^ ^20^ ^]^ NHS‐activated PMMPs underwent two 30 min shaking in 0.2% ammonia to facilitate NHS release. The concentration of free NHS in the combined solvents was quantified using LC‐MS on a hydrophilic interaction liquid chromatography (HILIC) column. Based on the standard curve in Figure 3D, the NHS ester density on the PMMP surface was calculated to be 1117.8 µmol·g⁻¹. In comparison to commercially available NHS‐activated magnetic microspheres, the coupling efficiency of PMMPs significantly exceeds that of NHS‐activated magnetic polymer microspheres (Magploy) when conjugating deoxycholic acid after cystamine modification. PMMPs also offer advantages over NHS‐activated magnetic agarose microspheres (Magarose), including reduced reagent dosage (Figure 3E). The PMMPs synthesized in this study demonstrate not only simplicity and convenience in their preparation but also utilize readily accessible raw materials. Four PMMPs were synthesized using two types of silica‐coated SiOH‐ Fe_3_O_4_‐MNPs (250 and 500 nm) and two types of uncoated Fe_3_O_4_‐MNPs (200 and 300 nm), all of which demonstrated excellent magnetic separation properties. SEM images revealed that all variants exhibited similar particle sizes and displayed a fluffy, non‐spherical morphology (Figure S3, Supporting Information). Further analysis revealed comparable ligand coupling abilities among the variants (Figure 3F), facilitating the straightforward and feasible mass production of PMMPs in the laboratory for subsequent investigations.

### 2.4 Synthesis and Characterization of Magnetic Probes on PMMPs

Short‐chain fatty acids (SCFAs) and bile acids (BAs) represent two of the most important classes of metabolites produced by gut microbiota.^[^ ^29^, ^30^ ^]^ Studies have shown a direct correlation between fecal SCFA profiles and host physiology, making them reliable, non‐invasive biomarkers for various disease states.^[^ ^31^ ^]^ LC‐MS technology offers advantages such as high sensitivity and selectivity, making it the most widely used platform for metabolite detection in biological samples.^[^ ^32^ ^]^ In contrast to metabolites like BAs, the hydrophilicity of SCFAs presents challenges related to retention and separation on chromatographic columns during LC‐MS analysis. Chemical derivatization can reduce the polarity and volatility of small molecule metabolites, which improves chromatographic separation and enhances the ionization efficiency of targeted analytes in LC‐MS, significantly boosting detection sensitivity.^[^ ^33^ ^]^ However, many derivatization processes are complex and time‐consuming, which can considerably increase both workload and analysis time.^[^ ^30^ ^]^ Thus, there is a growing need for a valuable and convenient analytical tool to facilitate rapid and robust analysis of SCFAs in biological research.

**Figure 4:**
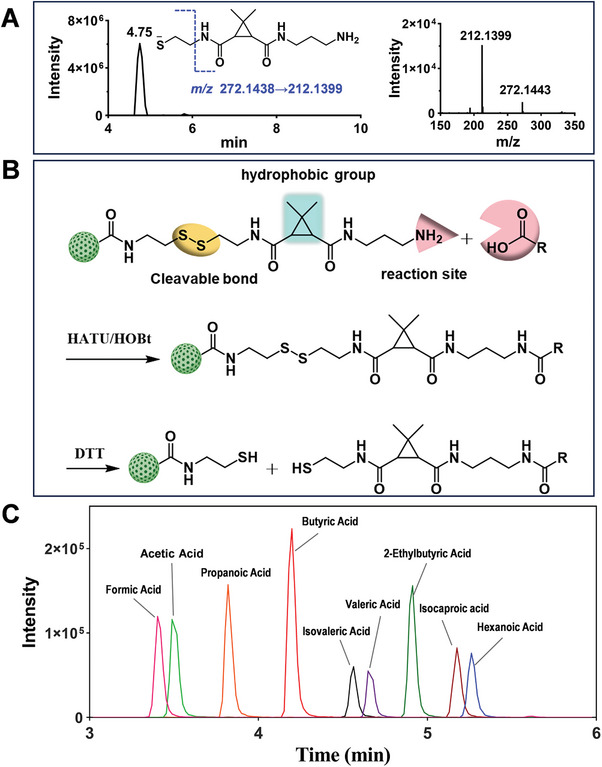
A) HPLC chromatograms and mass spectra of cleaved probe compounds. B) Amine‐terminated selective probes immobilized on PMMPs for the capture of carboxylic compounds and their cleavage to release modified metabolites. C) Chromatogram of 9 standard SCFA conjugates analyzed using UPLC‐MS.

NHS‐activated PMMPs were modified with amine‐terminated selective probes featuring cleavable bonds and hydrophobic tags to produce magnetic chemical probes. This design facilitates the straightforward capture of metabolites from complex matrix samples and enables their release under mild conditions (Figure S4, Supporting Information). UPLC‐MS analyses demonstrated that the cystamine scaffold is rapidly and quantitatively cleaved within 5 min (**Figure** **4**A). Additional cleavage experiments were conducted at each step to confirm the successful progression of the chemical reactions. The modification of cystamine and the activation of carboxylic groups via EDC/NHS followed previously established protocols.^[^ ^4^ ^]^ Two carboxylic compounds, deoxycholic acid, and butyric acid, were employed as target analytes to optimize the coupling steps involving caronic anhydride and DAP (Figure S5, Supporting Information), thereby maximizing the efficiency of each synthesis step following immobilization on PMMPs. The stability of the magnetic probe was further investigated, and it was found to remain stable for up to 14 days. However, prolonged storage (≈180 days) led to a slight reduction in its coupling efficiency (Figure S6, Supporting Information), which may be attributed to the agglomeration of the magnetic material.

As shown in Figure 4B, in the presence of the cross‐linking agents HATU and 1‐hydroxybenzotriazole (HOBt), amine‐terminated chemical probes immobilized on PMMPs capture carbonyl compounds and then rapidly release the analyte conjugates from PMMPs by reducing the disulfide bonds. The increase in molecular weight of 255.1405 Da resulting from the probe labeling procedure allows for the analysis of small and volatile metabolites, such as acetic acid and propionic acid. In addition, the introduction of a hydrophobic moiety transforms polar metabolites into hydrophobic derivatives with reduced polarity, which enhances their retention on reversed‐phase LC. Consequently, all 14 carboxylates were successfully captured and detected using UPLC‐MS equipped with a C18 column (Figure S7, Supporting Information). Notably, 9 SCFAs with high polarity, including formic acid, acetic acid, and propionic acid, were effectively separated (Figure 4C), enabling the simultaneous analysis of carboxylate metabolites, including SCFAs and free BAs, in a single run. Detailed results of the analysis of 14 carboxylate conjugates using UPLC‐MS are presented in Table S1 (Supporting Information).

### 2.5 Analysis of SCFAs in Cecal Content Samples

The magnetic probes immobilized on PMMPs were utilized in cecal content samples to further investigate their potential in real biological contexts. Due to the varying matrix environments and the presence of metabolites such as amines, the effective capture of carboxylated compounds by probes is greatly hampered. Methods used for capturing carboxylated standards are nearly impossible to detect target peaks from cecal contents. Therefore, further optimization in biological samples is essential to improve capture efficiency and analytical sensitivity. First, the pre‐activation of carboxylated metabolites with HATU in biological samples is undesirable because the carboxyl groups might preferentially react with amine metabolites. During the capture process, the biological samples and magnetic probes were thoroughly mixed before the final addition of HATU. The impact of HATU/HOBt and diisopropylethylamine (DIPEA) concentrations in the matrix of cecal contents was further examined using five carboxylated metabolites as target analytes. The optimized conditions of 0.5 mm HATU/HOBt and 5% DIPEA (*v*/*v*) were established for subsequent analysis of biological samples (Figure S8, Supporting Information). The reproducibility of the newly developed analytical method was evaluated through the independent preparation and analysis of five identical cecal content samples of rats. A total of 20 carboxylated metabolites, comprising 7 SCFAs and 5 bile acids, along with 8 isomers identified as potential bile acid derivatives, were characterized. These 20 peak areas were subsequently utilized to evaluate the method's reproducibility (Table S2, Supporting Information). The relative standard deviations (RSDs) of the peak areas for the 20 carboxylated metabolites detected were below 10%, indicating good reproducibility of the analytical method.

**Figure 5:**
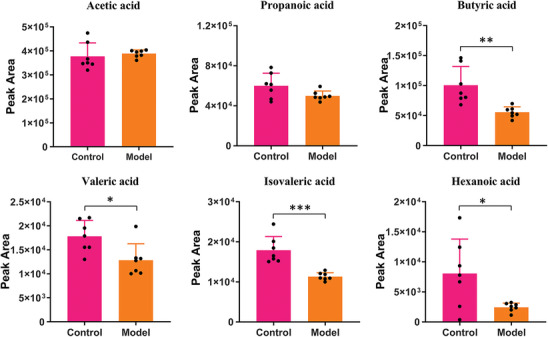
Analysis results of SCFA levels in the cecal contents of control and model rats. Data are expressed as mean ± SD (*n* = 7). Student's *t*‐test was used in statistical analysis. ^*^ *p* < 0.05; ^**^ *p* < 0.01; and ^***^ *p* < 0.001.

Depression is a prevalent and debilitating neuropsychiatric disorder that significantly impairs the quality of life of affected individuals.^[^ ^34^ ^]^ Studies have demonstrated a significant correlation between the concentration of SCFAs in the cecal contents and depression.^[^ ^35^, ^36^ ^]^ Consequently, the Chronic Unpredictable Mild Stress (CUMS) method was used to establish the depressed rat model, allowing for the analysis of SCFA concentration differences between depressed (*n* = 7) and wild‐type rats (*n* = 7). The detection sensitivity of the probe‐labeled derivatization strategy was compared with non‐derivatization methods, including LC‐MS and GC‐MS, for the analysis of six SCFAs. The results demonstrated that the sensitivity of the six SCFAs was significantly enhanced to the nanomolar level following labeling derivatization (Table S3, Supporting Information). The peak areas of SCFAs in these cecal content samples are detailed in Tables S4 and S5 (Supporting Information). As shown in **Figure** **5**, significant reductions of butyric acid, valeric acid, isovaleric acid, and hexanoic acid were observed in the model group compared to the control group. These findings are consistent with the absolute quantification results from GC‐MS analysis of the same model animal samples,^[^ ^37^ ^]^ highlighting the reliability of the analytical method. The observed reductions in SCFA metabolites in the model group may be attributed to changes in gut microbiota composition induced by depression.

## 3 Conclusion

In this study, polyurea particles with NHS‐ester were efficiently synthesized via a straightforward diamine‐diisocyanate polyurea process in the presence of NHS. The newly developed high‐density NHS‐activated PMMPs serve as a versatile and high‐performance magnetic material, providing a valuable tool in chemical biology for the separation and analysis of metabolites in biological samples. The multifunctional chemical probes immobilized on the PMMPs feature cleavable groups, hydrophobic regions, and terminal amino reactive sites, enabling the specific capture of carboxylated compounds in complex matrices, particularly SCFAs. These probes can subsequently release target analytes through DTT cleavage of the disulfide‐bonded linker. This chemoselective extraction strategy facilitates LC‐MS analysis of volatile SCFAs in the cecal contents, revealing significant reductions in butyrate, pentanoate, isopentanoate, and hexanoate levels in depressed rats. In summary, the developed one‐pot method for synthesizing NHS‐activated PMMPs demonstrates high coupling efficiency and cost‐effectiveness, making it a promising immobilization platform for various chemical probes and providing a convenient tool for analyzing complex biological samples.

## 4 Experimental Section

Experimental section and any associated references are available in the Supporting Information.

## Conflict of Interest

The authors declare no conflict of interest.

## Author Contributions

S.L. conceived the project, designed the study, and drafted the paper. M.Y. assisted in the experiments. X.Y.L. collected the experimental data. J.L. assisted in the method development. Z.M.Z. conceived and supervised the project. All authors have given approval to the final version of the manuscript.

## Supporting information



Supporting Information

## Data Availability

The data that support the findings of this study are available from the corresponding author upon reasonable request.
