# Supplementary material for: One‐Pot Construction of NHS‐Activated Magnetic Particles for Chemoselective Capture of Carboxyl Metabolites
Source: Adv Sci (Weinh). 2025 Feb 11;12(13):2413830. doi: 10.1002/advs.202413830 (PMC11967832; doi:10.1002/advs.202413830)
Supplement: Supplementary file 1 — Supporting Information [file ADVS-12-2413830-s001.docx]

**Supplementary data**

**One-pot Construction** **of NHS-****Activated Magnetic Particles for Chemoselective Capture of Carboxyl Metabolites**

*Shuai Liu ^a^, Meng Yu ^a^,* *Xin-Yao Luo ^a^, Jie Liu ^b^, Zhong-Mei Zou ^a,^* *

*^a^* State Key Laboratory of Bioactive Substance and Function of Natural Medicines, Institute of Medicinal Plant Development, Chinese Academy of Medical Sciences ＆ Peking Union Medical College, Beijing 100193, China

*^b^* Tianjin University of Traditional Chinese Medicine, Tianjin, 300193, China

*Corresponding author:

Zhong-Mei Zou, Institute of Medicinal Plant Development, Chinese Academy of Medical Sciences and Peking Union Medical College, Beijing 100193, China, Tel: +861057833290, E-mail: zmzou@implad.ac.cn.

**Table of Contents**

[Supplemental Experimental Procedures 4](#_Toc187871030)

[Materials 4](#_Toc187871031)

[Preparation of F_3_O_4_ nanoparticles 4](#_Toc187871032)

[Synthesis of the NHS-activated PMMPs 4](#_Toc187871033)

[Synthesis of magnetic probes 5](#_Toc187871034)

[Determination of NHS-ester amount on PMMPs 5](#_Toc187871035)

[Characterization of PMMPs and polymer structure 6](#_Toc187871036)

[Animals 6](#_Toc187871037)

[Biological Sample preparation 6](#_Toc187871038)

[Capture of the standard of bile acids and short-chain fatty acids 7](#_Toc187871039)

[Capture of carboxylic compounds in cecal contents 7](#_Toc187871040)

[Analysis of SCFA target analytes labeled with probes 7](#_Toc187871041)

[Analysis of SCFAs using GC-MS^[3]^ 8](#_Toc187871042)

[Statistical Analysis 8](#_Toc187871043)

[Supplemental Figures 10](#_Toc187871044)

[Figure S1. SEM images of polymer particles synthesized in DMSO without NHS. The molar ratio of DAP and HDI was 1:1.5. Magnetic polymer particles were prepared using commercial SiOH-Fe_3_O_4_-MNPs (A) and laboratory synthesized Fe_3_O_4_-MNPs (B) as magnetic core. 10](#_Toc187871045)

[Figure S2. The chemical reaction scheme for the coupling of amine ligands to carboxyl groups. The carboxyl group is activated with EDC/NHS (step 1) followed by covalent attachment of the ligand by its primary amine (step 2). 10](#_Toc187871046)

[Figure S3. SEM images of PMMPs made from SiOH-Fe_3_O_4_-MNPs and Fe_3_O_4_-MNPs with particle sizes of 200-500 nm. 11](#_Toc187871047)

[Figure S4. The synthesis route of magnetic probes and strategies for chemoselective capture of carboxylic compounds. 11](#_Toc187871048)

[Figure S5. The optimization of (A) concentration of caronic anhydride, (B) DAP concentration, and (C) triethylamine ratio as well as (D) reaction time. 12](#_Toc187871049)

[Figure S6. Impact of storage duration on the efficiency of magnetic probes in capturing butyric acid and deoxycholic acid. 12](#_Toc187871050)

[Figure S7. Extracted ion chromatogram of 14 carboxylate conjugates analyzed by UPLC-MS. 13](#_Toc187871051)

[Figure S8. Optimization of HATU/HOBt and DIPEA concentrations in cecal samples. 13](#_Toc187871052)

[Supplemental Tables 14](#_Toc187871053)

[Table S1. Results of the analysis of 14 carboxylate conjugates using UPLC-MS. 14](#_Toc187871054)

[Table S2. Reproducibility of 20 carboxylated metabolites across 5 replicate samples. 15](#_Toc187871055)

[Table S3. Sensitivity of unlabeled and labeled SCFAs. 16](#_Toc187871056)

[Table S4. The peak areas of SCFAs conjugated compounds detected in the cecal samples of wild rats (n=7). 16](#_Toc187871057)

[Table S5. The peak areas of SCFAs conjugated compounds detected in the cecal samples of depression model rats (n=7). 16](#_Toc187871058)

## Supplemental Experimental Procedures

### Materials

Hexamethylene diisocyanate (HDI), 1,4-diisocyanatobutane (DICB), 4,4'-methylenediphenyl diisocyanate (MDI), m-xylylene diisocyanate (XDI), 1,4-phenylene diisocyanate (PPDI), 1,3-diaminopropane (DAP), N-hydroxysuccinimide (NHS), diisopropylethylamine (DIPEA), O-(7-azabenzotriazol-1-yl)-N,N,N',N'-tetramethyluronium hexafluorophosphate (HATU), triethylamine (TEA), 1-(3-dimethylaminopropyl)-3-ethylcarbodiimide (EDC), 1-hydroxybenzotriazole (HOBt), N, N-dimethylformamide (DMF), dimethyl sulfoxide (DMSO), 2-morpholinoethanesulfonic acid (MES), cystamine dihydrochloride, were purchased from Aladdin Reagent Co. (Shanghai, China). LC-MS grade acetonitrile, methanol, and water were purchased from Thermo Fisher Scientific International, Inc. (Pittsburgh, PA, USA). Fluorescein 5 (6)-isothiocyanate (FITC) were purchased from Sigma-Aldrich (St. Louis, MO, USA). Acetic acid, propanoic acid, butyric acid, isobutyric acid, valeric acid, isovaleric acid, hexanoic acid and 2-ethylbutyric acid were purchased from Macklin Inc. (Macklin, Shanghai, China; purity≥ 98%). Cholic acid, chenodeoxycholic acid, lithocholic acid, deoxycholic acid, hyodeoxycholic acid were purchased from Cayman Chemical Company (Michigan, USA).

### Preparation of F_3_O_4_ nanoparticles

The magnetic Fe_3_O_4_ nanoparticles were prepared by the solvothermal method. Initially, 1.08 g of FeCl_3_·6H₂O was dissolved in a mixture of 14 mL of ethylene glycol and 26 mL of diethylene glycol. Subsequently, 4.00 g of sodium acetate (NaAc) was added, and the resulting mixture was stirred continuously for 1 hour. The solution was then transferred to a reaction vessel and heated at 200℃for 15 hours, followed by cooling to room temperature. The resulting products were washed multiple times with ethanol and deionized water, and then dried at 50 °C for 15 hours.

### Synthesis of the NHS-activated PMMPs

The synthesis of magnetic polyurea particles was conducted in DMF solution. Specifically, 1 mg of purified F_3_O_4_ nanoparticles, 11.5 mg of NHS, and 1 *μ*L of diaminopropane (DAP) were added to 0.6 mL of DMF. Subsequently, 400 *μ*L of a 1.5% hexamethylene diisocyanate (HDI) solution was rapidly introduced and thoroughly mixed. The reaction mixture was shaken at 25 °C for 60 min. The magnetic polyurea particles were then collected using a magnet and washed with DMF to remove unreacted components. The prepared PMMPs were stored in DMF for future use.

### Synthesis of magnetic probes

A 1 M cystamine solution was prepared by dissolving 2.25 g of cystamine hydrochloride in 9 mL of MES buffer (0.1 M, containing 0.5% Tween-20) along with 1 mL of triethylamine. Typically, 50 mg of PMMPs were dispersed in 10 mL of the cystamine dihydrochloride solution and shaken for 30 min at 25 °C. The product was separated using a magnet, washed with water and DMF, and then re-dispersed in 10 mL of DMF. Subsequently, 1.4 g of phosgene was added to the suspension, which was shaken at 50 °C for 10 h Afterward, the separated products were washed with DMF and re-dispersed in 10 mL of dichloromethane. To this suspension, 1.92 g of EDC and 1.15 g of NHS were added, and the mixture was shaken at 25 °C for 12 h. The product was then separated, washed with DMF, and 10 mL of DAP solution (v/v, 1:5, dissolved in DMF) was added. The mixture was shaken at 25 °C for 1 h. Finally, the prepared magnetic probes were separated, washed with DMF and DMSO, and re-dispersed in DMSO for further use.

### Determination of NHS-ester amount on PMMPs

NHS-ester is unstable in alkaline solutions and hydrolyzes rapidly^[1]^. Therefore, the cleaned PMMPs (n=3) were added to 0.2% ammonia solution and shaken for 30 min to release free NHS, this process was repeated twice to combine the resulting solutions. The analysis of NHS was conducted using the Waters ACQUITY Ultra Performance Liquid Chromatography (UPLC) system, equipped STNAPY G2 HDMS system (Waters Corp., Manchester, UK) in negative mode. A Waters ACQUITY UPLC BEH HILIC column (2.1 mm×100 mm, 1.7 *μ*m particle size, Waters Corp., Milford, MA, USA) was used for the separation of the strongly polar NHS. The binary mobile phase consisted of acetonitrile (A) and 10 mM ammonium formate aqueous solution containing 0.05% NH_3_·H_2_O (B) with a flow rate of 0.2 mL/min. The gradient elution profile was as follows: 0-2 min at 85% B, 5-8 min at 80% B, 10 min at 75% B, 11-12 min at 60% B, 13-15 min at 85% B, followed by a hold at 90% B for 3 min to equilibrate the column. The temperatures of the sample tray and column oven were maintained at 4 and 35 °C, respectively, with an injection volume of 5 *μ*L.

### Characterization of PMMPs and polymer structure

The morphology of PMMPs and polyurea material was examined using a Cold Field Emission Scanning Electron Microscope (SEM, SU-8010, Hitachi, Japan). The chemical structure of the polyurea material was characterized by Fourier transform infrared spectra (FTIR, IRAffinity-1, Shimadzu, Japan), with scans performed from 400 to 4000 cm^–1^. The conformation of the polyurea material was investigated using solid-state ^13^C NMR (AVANCE III 600 MHz spectrometer, Bruker, USA). The magnetic properties of the PMMPs were measured with a Vibrating Sample Magnetometer (VSM, LakeShore 7404, USA).

### Animals

Fourteen male Sprague-Dawley (SD) rats (180-220 g) were purchased from Beijing Vital River Laboratory Animal Technology Co., Ltd. (Beijing, China). All rats were housed in the animal experimentation center of the Institute of Medicinal Plant Development. The rats were housed under specific conditions: room temperature was set at 20-25 °C, relative humidity was maintained at 50 ±5%, and illumination followed a 12:12 h light-dark cycle. Five rats were housed in each cage to ensure optimal SPF cleanliness. All experimental procedures followed the ARRIVE guidelines 12 and were approved by the Animal Care and Use Committee of the Institute of Material Plant Development, CAMS & PUMC (license No. SYXK (Jing) 2017-0020).

Following 3 days of adaptive feeding, the open field test (OFT) and sucrose preference test (SPT) were performed. The rats were randomly assigned to two groups, each consisting of seven animals, based on their behavioral scores and body weights. These groups included a control group and a model group. Each group received the respective treatment via gavage for a duration of 35 days.

The CUMS method was used to establish the depressed rat model, following previously reported procedures^[2]^. At the end of the experiment, all rats were fasted for 12 hours and sacrificed under anesthesia with 1% pentobarbital sodium. Cecal contents were collected, snap frozen in liquid nitrogen, and transferred to -80 °C for storage.

### Biological Sample preparation

Cecal content samples stored at -80 °C were thawed on ice. Approximately 100 mg was weighed and transferred to a 1.5 mL centrifuge tube, followed by the addition of 1 mL of acetonitrile. The mixture was vortexed at 4 °C for 5 minutes, sonicated for 15 min, and then centrifuged at 13,000 rpm for 15 min. The supernatant was carefully aspirated for subsequent use.

### Capture of the standard of bile acids and short-chain fatty acids

Bile acids and short-chain fatty acid standards were dissolved in DMSO, and serial dilutions were prepared to generate a range of control and mixed solutions at various concentrations.

2mg of magnetic probes were dispersed in 968 *μ*L of DMSO and mixed with 10 *μ*L of standard solution. Subsequently, 2 *μ*L of DIPEA and 10 *μ*L of freshly prepared HOBt solution (100 mM, dissolved in DMSO) were added and mixed thoroughly. A rapid addition of 10 *μ*L of freshly prepared HATU solution (100 mM, dissolved in DMSO) followed, and the mixture was shaken at 25 °C for 1 h. The probes were then washed twice, first with 50% aqueous acetonitrile and then with pure acetonitrile, before being re-dispersed in 985 *μ*L of 50% aqueous acetonitrile. Following this, 15 *μ*L of 0.1 M DTT aqueous solution was added, and the target analytes were released by shaking at 25 °C for 15 min. The supernatant was collected using magnetic separation and centrifuged at 13,000 rpm for 15 min at 4 °C prior to UPLC-Q-TOF-MS detection.

### Capture of carboxylic compounds in cecal contents

2mg of magnetic probes were dispersed in 890 *μ*L of DMSO and mixed with 50 *μ*L of cecal content extract. Subsequently, 50 *μ*L of DIPEA and 5 *μ*L of freshly prepared HOBt solution (100 mM, dissolved in DMSO) were added and mixed thoroughly. A rapid addition of 5 *μ*L of freshly prepared HATU solution (100 mM, dissolved in DMSO) followed, and the mixture was shaken at 25 °C for 1 h. The probes were then washed twice, first with 50% aqueous acetonitrile and then with pure acetonitrile, before being re-dispersed in 985 *μ*L of 50% aqueous acetonitrile. Following this, 15 *μ*L of 0.1 M DTT aqueous solution was added, and the target analytes were released by shaking at 25 °C for 15 min. The supernatant was collected using magnetic separation and centrifuged at 13,000 rpm for 15 min at 4 °C prior to UPLC-Q-TOF-MS detection.

### Analysis of SCFA target analytes labeled with probes

The UPLC-Q-TOF-MS/MS system was utilized for the analysis of short-chain fatty acids (SCFAs) and bile acids in rat cecal contents. Following labeling with the probe, the molecular weight and hydrophobicity of SCFAs increased, necessitating separation on a Waters ACQUITY BEH C18 column (1.7 µm, 2.1 mm × 100 mm). The mobile phase comprised (A) 0.1% formic acid in water and (B) 0.1% formic acid in acetonitrile. The gradient elution profile was as follows: 0-3 min/5-40% B, 3-5 min/40% B, 5-6 min/40-42% B, 6-8 min/42-50% B, 8-10 min/50-98% B, 10-12 min/95% B, 12-12.5 min/5% B, followed by a 2.5 min equilibration at 5% B. The sample tray and column oven temperatures were maintained at 4 °C and 35 °C, respectively, with a flow rate of 0.2 mL/min and an injection volume of 2 *µ*L.

HRMS analysis was conducted using a STNAPY G2 HDMS system (Waters Corp., Milford, MA, USA) in negative ion mode, equipped with an electrospray ionization (ESI) source. The capillary voltage, sample cone voltage, and extraction cone voltage were set to 1.5 kV, 40 V, and 4 V, respectively. The desolvation gas, nitrogen, was maintained at a flow rate of 600 L·h⁻¹ and a temperature of 400 °C, while the cone gas flow rate was set to 50 L·h⁻¹ and the source temperature to 100 °C. The scan time and scan time interval were adjusted to 0.5 s and 0.02 s, respectively. Mass-to-charge ratios were recorded from m/z 50 to *m/z* 1200 in centroid mode, normal resolution, and extended dynamic range. Leucine enkephalin (LE) was utilized as the lock mass at *m/z* 554.2615 for the negative ion mode.

### Analysis of SCFAs using GC-MS^[3]^

Using ethyl acetate as the solvent, a mixture of standards including acetic acid, propanoic acid, butyric acid, isobutyric acid, valeric acid, isovaleric acid and hexanoic acid was produced. The internal standard concentration in the combination was matched to that of the samples. The data were collected using Thermo Field TRACE GC ULTRA ISQ and HIP FFAP capillary column (30 m ×0.25 mm×0.25 µm, Agilent J& W Scientific, Folsom, CA, USA). Nitrogen was used as the carrier gas at a flow rate of 1 mL/min. Scanning mode was SIM mode. The initial temperature was 80 ℃, programmed at 40 ℃/min to 120 ℃, then at 10 ℃/min to 200 ℃, and at 10 ℃/min to 230 ℃ (held for 1 min at each temperature).

### Statistical Analysis

The experimental data were statistically analyzed and plotted using GraphPad Prism 9 software (GraphPad Software, San Diego, USA) and Origin 2022 software (Origin Laboratories, Northampton, MA). Student's test was employed to assess differences between two experimental groups, with a *p*-value <0.05 deemed statistically significant. MassLynx (version 4.1; Waters Corp., Milford, MA, USA) was used to process the MS data. Detailed statistical methods are outlined in the figure legends.

## Supplemental Figures


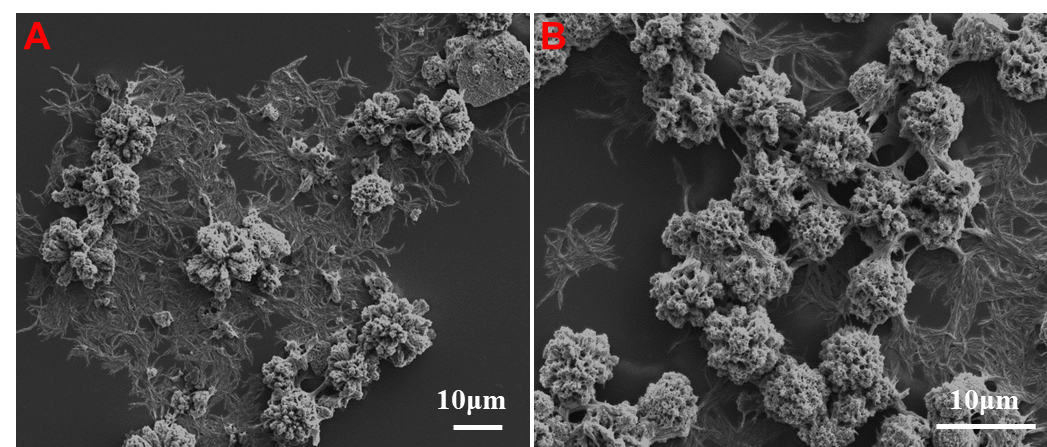


### Figure S1. SEM images of polymer particles synthesized in DMSO without NHS. The molar ratio of DAP and HDI was 1:1.5. Magnetic polymer particles were prepared using commercial SiOH-Fe_3_O_4_-MNPs (A) and laboratory synthesized Fe_3_O_4_-MNPs (B) as magnetic core.


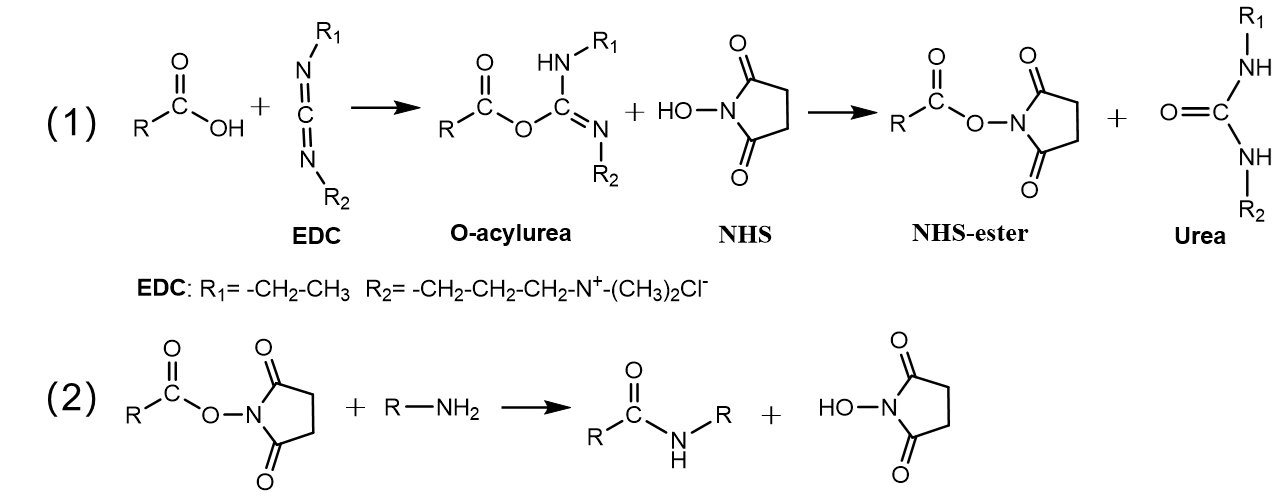


### Figure S2. The chemical reaction scheme for the coupling of amine ligands to carboxyl groups. The carboxyl group is activated with EDC/NHS (step 1) followed by covalent attachment of the ligand by its primary amine (step 2).


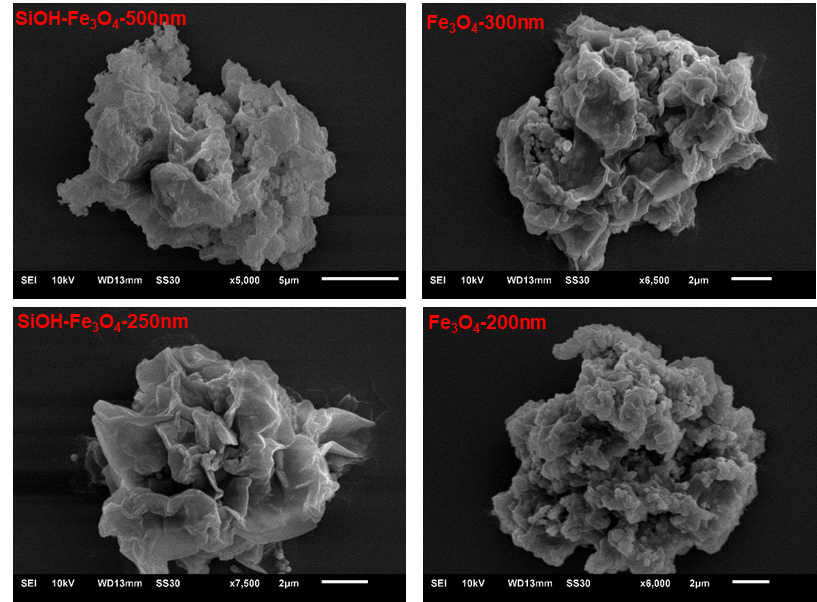


### Figure S3. SEM images of PMMPs made from SiOH-Fe_3_O_4_-MNPs and Fe_3_O_4_-MNPs with particle sizes of 200-500 nm.

### Figure S4. The synthesis route of magnetic probes and strategies for chemoselective capture of carboxylic compounds.


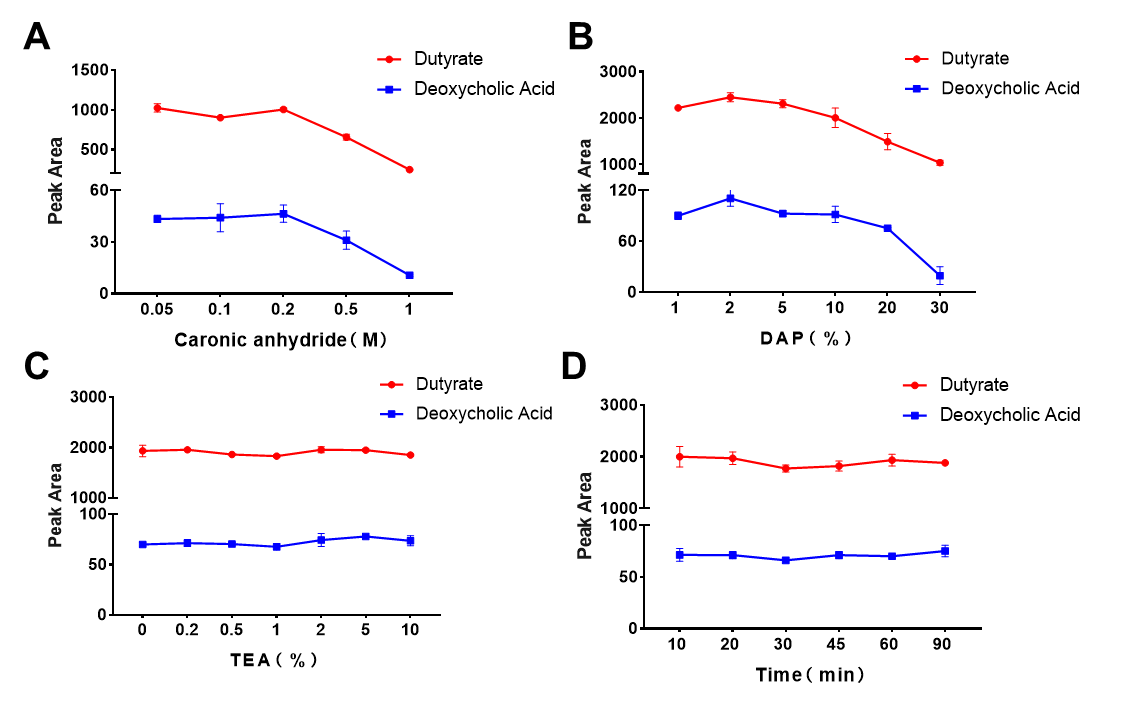


### Figure S5. The optimization of (A) concentration of caronic anhydride, (B) DAP concentration, and (C) triethylamine ratio as well as (D) reaction time.

### Figure S6. Impact of storage duration on the efficiency of magnetic probes in capturing butyric acid and deoxycholic acid.


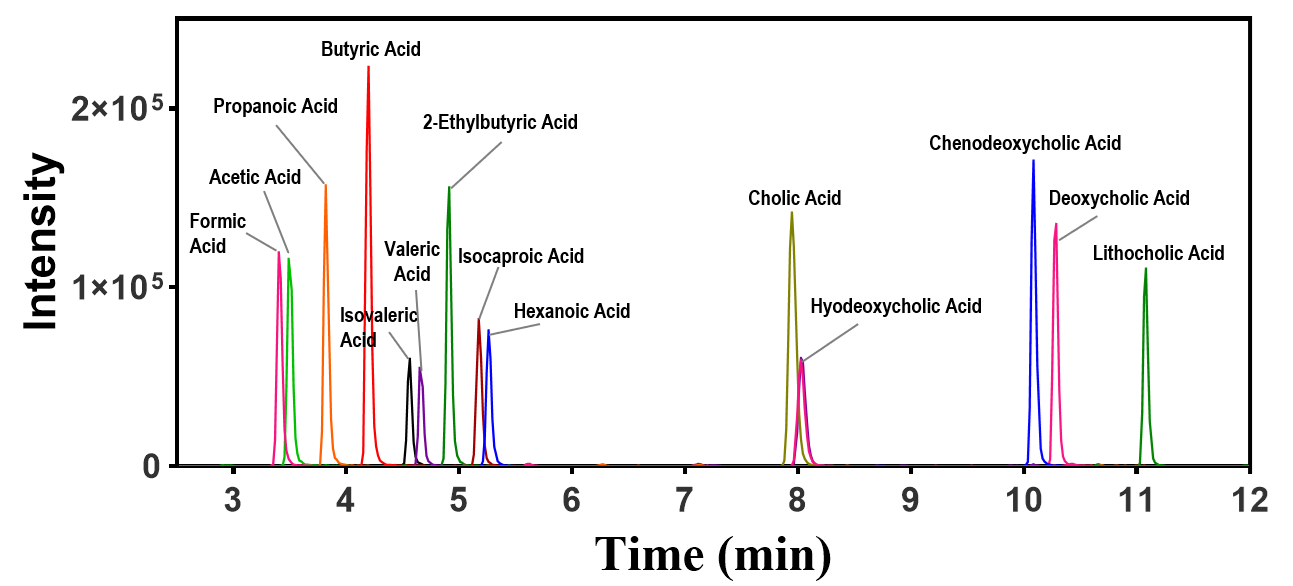


### Figure S7. Extracted ion chromatogram of 14 carboxylate conjugates analyzed by UPLC-MS.


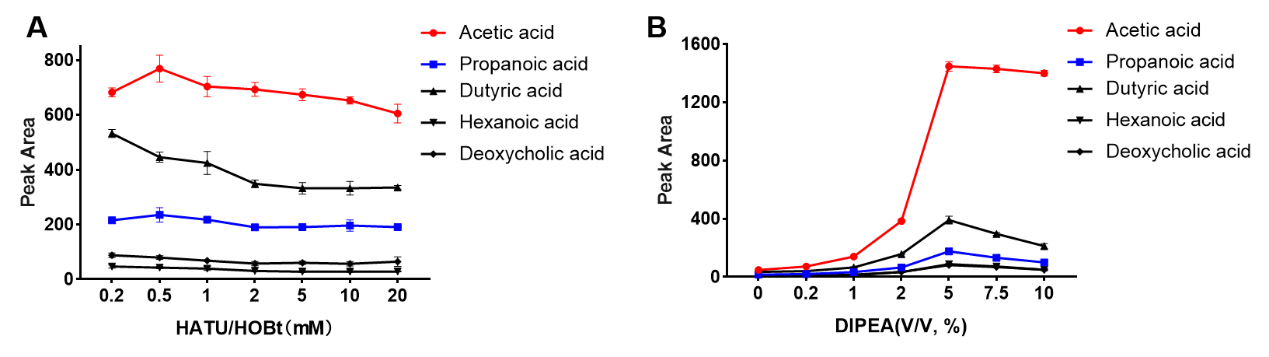


### Figure S8. Optimization of HATU/HOBt and DIPEA concentrations in cecal samples.

## Supplemental Tables

### Table S1. Results of the analysis of 14 carboxylate conjugates using UPLC-MS.

| **Compound** | **Theoretical m/z**  **[M-H]-** | **Observed m/z**  **[M-H]-** | **Error**  **(ppm)** | **Retention time(min)** |
| --- | --- | --- | --- | --- |
| Formic acid | 300.1387 | 300.1407 | 6.7 | 3.40 |
| Acetic acid | 314.1544 | 314.1567 | 7.3 | 3.49 |
| Propanoic acid | 328.1700 | 328.1702 | 0.6 | 3.82 |
| Butyric acid | 342.1857 | 342.186 | 0.9 | 4.2 |
| Valeric acid | 356.2013 | 356.1994 | 5.3 | 4.65 |
| Isovaleric acid | 356.2013 | 356.1994 | 5.3 | 4.56 |
| Hexanoic acid | 370.2170 | 370.2159 | 3.0 | 5.26 |
| 2-Ethylbutyric acid | 370.2170 | 370.2159 | 3.0 | 4.91 |
| Isocaproic acid | 370.2170 | 370.2159 | 3.0 | 5.17 |
| Cholic acid | 662.4208 | 662.4186 | 3.3 | 7.95 |
| Chenodeoxycholic acid | 646.4259 | 646.4287 | 4.3 | 10.08 |
| Lithocholic acid | 630.4310 | 630.4281 | 4.6 | 11.08 |
| Deoxycholic acid | 646.4259 | 646.4287 | 4.3 | 10.28 |
| Hyodeoxycholic acid | 646.4259 | 646.4287 | 4.3 | 8.03 |

### Table S2. Reproducibility of 20 carboxylated metabolites across 5 replicate samples.

| **Compound** | **Retention time**  **(min)** | **Theoretical m/z**  **[M-H]-** | **Observed**  **m/z**  **[M-H]-** | **Error**  **(ppm)** | **Mean peak**  **area** | **RSD**  **(%)** |
| --- | --- | --- | --- | --- | --- | --- |
| Formic acid | 3.4 | 300.1387 | 300.1396 | 3.00 | 213767 | 3.53% |
| Acetic acid | 3.49 | 314.1544 | 314.1563 | 6.05 | 371229 | 2.58% |
| Propanoic acid | 3.8 | 328.17 | 328.1669 | 9.45 | 79520 | 9.98% |
| Butyric acid | 4.18 | 342.1857 | 342.1867 | 2.92 | 106535 | 5.01% |
| Valeric acid | 4.65 | 356.2013 | 356.2045 | 8.98 | 14424 | 3.83% |
| Isovaleric acid | 4.55 | 356.2013 | 356.2045 | 8.98 | 15119 | 6.19% |
| Hexanoic acid | 5.26 | 370.217 | 370.2178 | 2.16 | 3914 | 4.26% |
| Cholic acid isomer | 5.48 | 662.4208 | 662.4155 | 8.00 | 1545 | 3.20% |
| Cholic acid isomer | 5.67 | 662.4208 | 662.4155 | 8.00 | 5145 | 8.23% |
| Cholic acid isomer | 5.89 | 662.4208 | 662.4155 | 8.00 | 4028 | 1.59% |
| Cholic acid isomer | 6.17 | 662.4208 | 662.4155 | 8.00 | 8418 | 3.43% |
| Cholic acid | 7.93 | 662.4208 | 662.4155 | 8.00 | 3902 | 2.72% |
| Hyodeoxycholic acid isomer | 7.23 | 646.4259 | 646.425 | 1.39 | 1581 | 3.87% |
| Hyodeoxycholic acid | 8.01 | 646.4259 | 646.425 | 1.39 | 7328 | 1.88% |
| Hyodeoxycholic acid isomer | 9.02 | 646.4259 | 646.425 | 1.39 | 836 | 3.32% |
| Chenodeoxycholic acid | 10.07 | 646.4259 | 646.425 | 1.39 | 481 | 3.39% |
| Deoxycholic acid | 10.27 | 646.4259 | 646.425 | 1.39 | 13086 | 3.62% |
| Deoxycholic acid isomer | 10.53 | 630.431 | 630.4336 | 4.12 | 97 | 3.27% |
| Deoxycholic acid isomer | 10.64 | 630.431 | 630.4336 | 4.12 | 169 | 5.76% |
| Lithocholic | 11.08 | 630.431 | 630.4336 | 4.12 | 1225 | 4.79% |

### Table S3. Sensitivity of unlabeled and labeled SCFAs.

| **SCFAs** | **Unlabeled** | | **labeled** |
| --- | --- | --- | --- |
|  | **LOD**  **LC-MS（mM）** | **LOD**  **GC-MS（μM）** | **LOD**  **LC-MS（nM）** |
| Acetic acid | N/A | 0.50 | 1.75 |
| Propanoic acid | N/A | 3.42 | 1.34 |
| Butyric acid | 1.09 | 0.15 | 1.09 |
| Valeric acid | 0.92 | 1.25 | 9.21 |
| Isovaleric acid | 0.91 | 2.51 | 0.91 |
| Hexanoic acid | 0.80 | 1.92 | 7.99 |

The UPLC-Q-TOF-MS/MS analysis of labeled and unlabeled SCFAs was conducted using identical chromatographic and mass spectrometric conditions.

### Table S4. The peak areas of SCFAs conjugated compounds detected in the cecal samples of wild rats (n=7).

| **Compound** | **1** | **2** | **3** | **4** | **5** | **6** | **7** |
| --- | --- | --- | --- | --- | --- | --- | --- |
| Acetic acid | 474245 | 436091 | 366275 | 346936 | 344713 | 350069 | 319764 |
| Propanoic acid | 72688 | 78293 | 46699 | 61223 | 43953 | 55543 | 61912 |
| Butyric acid | 146324 | 141031 | 87417 | 102694 | 80476 | 78854 | 68012 |
| Valeric acid | 21691 | 15534 | 15530 | 13016 | 17882 | 21513 | 19531 |
| Isovaleric acid | 20091 | 24405 | 15732 | 15038 | 15207 | 18427 | 16481 |
| Hexanoic acid | 12421 | 17309 | 7781 | 9320 | 2570 | 6661 | 350 |

### Table S5. The peak areas of SCFAs conjugated compounds detected in the cecal samples of depression model rats (n=7).

| **Compound** | **1** | **2** | **3** | **4** | **5** | **6** | **7** |
| --- | --- | --- | --- | --- | --- | --- | --- |
| Acetic acid | 381765 | 398950 | 395414 | 403739 | 378665 | 360654 | 400789 |
| Propanoic acid | 52472 | 48707 | 49355 | 43605 | 48428 | 47834 | 59229 |
| Butyric acid | 59771 | 69863 | 54605 | 41848 | 61063 | 49589 | 52162 |
| Valeric acid | 10025 | 13175 | 10160 | 12757 | 19866 | 13298 | 10649 |
| Isovaleric acid | 9982 | 10710 | 10937 | 10895 | 12867 | 11844 | 12056 |
| Hexanoic acid | 1122 | 3217 | 3043 | 1989 | 2337 | 2555 | 2834 |

[1] S. Liu, M. Zhang, Z. Lai, H. Tian, Y. Qiu, Z. Li, *ACS Applied Materials & Interfaces* **2022**, *14*, 32890.

[2] Z. Su, S. Li, G. Zou, C. Yu, Y. Sun, H. Zhang, Y. Gu, Z. Zou, *Journal of Pharmaceutical and Biomedical Analysis* **2011**, *55*, 533.

[3] X. Luo, M. Yu, H. Li, X. Kong, Z. Zou, X. Ye, *Carbohydrate Polymers* **2024**, 122779.
